# Preoperative Cervical Lymph Node Metastasis Prediction in Papillary Thyroid Carcinoma: A Noninvasive Clinical Multimodal Radiomics (CMR) Nomogram Analysis

**DOI:** 10.1155/2023/3270137

**Published:** 2023-03-09

**Authors:** Wenjuan Hu, Yuzhong Zhuang, Lang Tang, Hongyan Chen, Hao Wang, Ran Wei, Lanyun Wang, Yi Ding, Xiaoli Xie, Yaqiong Ge, Pu-Yeh Wu, Bin Song

**Affiliations:** ^1^Department of Radiology, Minhang Hospital, Fudan University, Minhang District, Shanghai, China; ^2^Department of Ultrasonography, Minhang Hospital, Fudan University, Minhang District, Shanghai, China; ^3^Department of Pathology, Minhang Hospital, Fudan University, Minhang District, Shanghai, China; ^4^GEHealthcare, Shanghai, China

## Abstract

This study aimed to evaluate the feasibility of applying a clinical multimodal radiomics nomogram based on ultrasonography (US) and multiparametric magnetic resonance imaging (MRI) for the prediction of cervical lymph node metastasis (LNM) in papillary thyroid carcinoma (PTC) preoperatively. We performed retrospective evaluations of 133 patients with pathologically confirmed PTC, who were assigned to the training cohort and validation cohort (7 : 3), and extracted radiomics features from the preoperative US, T2-weighted (T2WI),diffusion-weighted (DWI), and contrast-enhanced T1-weighted (CE-T1WI) images. Optimal subsets were selected using minimum redundancy, maximum relevance, and recursive feature elimination in the support vector machine (SVM). For LNM prediction, the radiomics model was constructed by SVM, and Multi-Omics Graph cOnvolutional NETworks (MOGONET) was used for the effective classification of multiradiomics data. Multivariable logistic regression incorporating multiradiomics signatures and clinical risk factors was used to generate a nomogram, whose performance and clinical utility were assessed. Results showed that the nine most predictive features were separately selected from US, T2WI, DWI, and CE-T1WI images, and 18 features were selected in the combined model. The combined radiomics model showed better performance than models based on US, T2WI, DWI, and CE-T1WI. In a comparison of the combined radiomics and MOGONET model, receiver operating curve analysis showed that the area under the curve (AUC) value (95% CI) was 0.84 (0.76–0.93) and 0.84 (0.71–0.96) for the MOGONET model in the training and validation cohorts, respectively. The corresponding values (95% CI) for the combined radiomics model were 0.82 (0.74–0.90) and 0.77 (0.61–0.94), respectively. The MOGONET model had better performance and better prediction specificity compared with the combined radiomics model. The nomogram including the MOGONET signature showed a better predictive value (AUC: 0.81 vs. 0.88) in the training and validation (AUC: 0.74vs. 0.87) cohorts, as compared with the clinical model. Calibration curves showed good agreement in both cohorts. The applicability of the clinical multimodal radiomics (CMR) nomogram in clinical settings was validated by decision curve analysis. In patients with PTC, the CMR nomogram could improve the prediction of cervical LNM preoperatively and may be helpful in clinical decision-making.

## 1. Introduction

In the past three decades, papillary thyroid cancer (PTC) incidence has continued to increase worldwide [1, 2]. Furthermore, PTC is the most commonly seen histology (89.1%) in thyroid cancer, and its incidence-based mortality rates continue to increase [3]. However, the mortality rates of PTC (0.3 per 100,000 in men and 0.5 per 100,000 in women) are very low [1]. Although PTC is an indolent tumor with a good prognosis, 30%–80% of cases may show cervical lymph node metastasis (LNM), which is extremely common (incidence rate, up to 41.3%) in papillary thyroid microcarcinoma [4]. Cervical LNM first develops in the central neck region corresponding to cervical level VI, and then in the lateral neck. Cervical LNM in PTC is an important factor determining the approach of surgery total thyroidectomy or lobectomy, bilateral or ipsilateral central node dissection (CND). It is also an independent factor influencing the risk of poor prognosis and local recurrence of PTC [5–7] and the most important factor predicting a high risk of lateral LNM [4]. Many PTC patients undergo procedures such as total thyroidectomy and CND to address the risk of cervical LNM, frequently resulting in overtreatment [8].

Although CND improves disease-specific survival and reduces local recurrence in cases of LNM [9], prophylactic CND has been reported to not improve long-term outcomes and is related to high hypoparathyroidism rates [10]. Given the increasing awareness about the substantial impact of PTC overdiagnosis, the guidelines of the American Thyroid Association (ATA) recommended and advocated thyroid lobectomy alone and active surveillance as initial treatments for low-risk PTC patients [11]. Furthermore, preoperative examinations should be improved to more accurately identify patients with high-risk PTC and provide individualized treatments.

Preoperative ultrasound (US) is useful for assessing lateral cervical LNM among patients with PTC [12]. However, its sensitivity for evaluating central cervical LNM is only 30%–50% [12, 13]. Contrast-enhanced US and elastosonography have been shown to be superior to conventional US [13, 14]. However, US evaluation is dependent on the operator and may not provide adequate visualization of deep anatomical structures and the structures that are obscured by the bone or air acoustically. Computed tomography (CT) shows greater sensitivity than the US for detecting central cervical LNM but lower sensitivity in predicting lateral cervical LNM. Thus, a noninvasive and effective approach to predict cervical LNM risk in PTC is essential for guiding diagnosis and treatment.

Risk analysis for the prediction of cervical LNM among patients with PTC has been proposed in several studies, and tumor size, location, extrathyroidal extension, and microcalcification were found to be independent risk factors of cervical LNM [4, 6, 7, 15]. Several cervical LNM prediction models were constructed by combining the above risk factors with US features [16–18]. Recently, radiomics has received great attention for its potential to facilitate accurate diagnosis. CT- and US-based radiomics approaches for predicting cervical LNM among patients with PTC have been reported in several studies [19–21]. A previous study confirmed that MRI-based radiomics showed good performance for preoperative cervical LNM prediction among patients with PTC, with an area under the receiver operating characteristic (ROC) curve (AUC) of 0.835 and 0.830 in the training and validation groups, respectively [22]. However, all of the aforementioned studies were conducted with a single imaging modality and used different methods for feature extraction and model construction. Combining features from multiple imaging modalities may further improve the performance of the radiomics model in preoperative cervical LNM prediction; however, there are few reports on the comparison between radiomics models based on different imaging modalities and multiple imaging modalities for predicting cervical LNM in PTC.

The widespread use of high-throughput technologies has led to the emergence of multiomics integrative analysis approaches. Researchers can obtain omics data at scale from different molecular levels such as the genome, transcriptome, proteome, interactome, epigenome, metabolome, liposome, and microbiome to advance the understanding of biological processes and molecular mechanisms.

Multi-Omics Graph cOnvolutional NETworks (MOGONET), a novel multiomics integrative method, was recently proposed by Wang et al. [23]. As a supervised algorithm based on a graph network, MOGONET outperforms other multiomics integration methods and is effective for multiomics data classification. In this study, we applied this method to a multiradiomics model based on US and multiparametric MRI data of thyroid neoplasms to predict cervical LNM among patients with PTC and compared the performance of the multiradiomics model constructed using MOGONET and support vector machine (SVM) followed by the construction of a predictive nomogram. We hypothesize that based on its superior performance in previous biomedical classifications, the MOGONET multiradiomics model may show a better ability to predict cervical LNM than that of traditional radiomics and clinical statistical models.

## 2. Materials and Methods

### 2.1. Patients

The ethics committee of Minhang Hospital, Fudan University School of Medicine, approved this study. All participants provided written informed consent before US and MRI examinations. This retrospective review was conducted using the data of 268 consecutive patients who presented with pathologically confirmed PTC at our hospital from January 1, 2017 to December 31, 2021. The inclusion criteria were as follows: (1) pathologically confirmed PTC; (2) receipt of neck lymph node dissection and preoperative MRI and US examinations; (3) no previous biopsy or surgery of the thyroid; and (4) no history of neck cancer or radiation therapy. The exclusion criteria were as follows: (1) maximum tumor diameter of <5 mm; (2) nonreceipt of lymph node dissection; (3) poor MRI quality; (4) measuring lines on US images; and (5) inconsistency between MR and US images. Finally, 133 patients were included; 58 (43.6%) and 75 (56.4%) patients did not have cervical LNM (non-LNM group) and had pathologically confirmed cervical LNM (LNM group), respectively. The patient selection flow chart is shown in Supplementary Figure 1.

### 2.2. MRI Data Acquisition

All MRI examinations were conducted on a 1.5T MRI scanner (EXCITE HD; GE) that was equipped with an eight-channel neck surface coil. The MRI protocols consisted of axial T2WI, axial DWI (*b* values: 0 and 800 s/mm^2^), and axial CE-T1WI. The detailed imaging parameters are listed in Supplementary Table 1. CE-T1WI was performed at 30, 60, 120, 180, 240, and 300 s after gadolinium (Magnevist, Bayer) injection at a flow rate of 3 ml/s. The images collected at the 300 s phase were used to extract features.

### 2.3. US Data Acquisition

US examinations were conducted on the Aplio i900 (Canon), Logic E9 (GE), and Toshiba 790A (Toshiba) ultrasound systems by radiologists (≥5 years' experience). The probe models included i6SVX1, ML6-15, and PLT-805AT, and the probe frequency was 5–15 MHz. All US examinations of the thyroid require the storage of the largest long-axis cross-section images of the lesions and other typical signs such as microcalcification and capsule invasion.

### 2.4. Tumor Segmentation

The largest long-axis cross-section images obtained using T2WI, DWI, and CE-T1WI were recorded from PACS by a radiologist (W. J. H.) (with >10 years of experience in MRI). The largest long-axis cross-section images of the tumor were recorded from PACS by a radiologist (H. Y. C.) (with >20 years of experience in US imaging of the thyroid). Tumor segmentation was performed using the ITK-SNAP software package (v3.8.0; https://www.itksnap.org), with manual delineation of regions of interest (ROIs) along the tumor edge on the largest long-axis cross-section from T2WI, DWI, CE-T1WI, and US images. A radiologist (W. J. H) performed MR image segmentation, while another radiologist (L. T.) (5 years' experience in the US) performed US image segmentation. The largest lesion in multifocal PTCs was evaluated for further analysis.

Subsequently, to evaluate feature reproducibility, a double-blind comparison of manual segmentation in 30 cases was performed by two pairs of radiologists (B. S. vs. W. J. H., L. T. vs. H. Y. C.). An intraclass correlation coefficient (ICCs) >0.75 was considered excellent reliability. Features with ICCs >0.75 in the first sketch of W. J. H and H. Y. C. were retained.

### 2.5. Feature Extraction and Selection

All images were normalized before the feature extraction procedure. The following features were extracted using the PyRadiomics package (3.0.1) [24] implemented in Python: gray-level co-occurrence matrix (GLCM), gray-level size zone matrix (GLSZM), gray-level run-length matrix (GLRLM), first-order statistics, Laplacian of Gaussian (LoG), and wavelet. Data were randomly divided into the training and validation groups at a ratio of 7 : 3. Two feature selection methods were applied. First, redundant features were eliminated, and features showing a high correlation with the labels were retained using minimum redundancy maximum relevance (mRMR). Twenty features were retained. Subsequently, the recursive feature elimination (RFE) algorithm was used to find a subset of predictors that could be used to produce an accurate model by the backward selection of predictors based on predictor importance ranking. The predictors were ranked, and the less important ones were sequentially eliminated before modelling.

### 2.6. Radiomics Model Construction and Nomogram Development

First, radiomics models based on US (US-radiomics), T2WI (T2WI-radiomics), DWI (DWI-radiomics), and CE-T1WI (CE-T1WI-radiomics) were constructed using SVM. Then, a multiparametric radiomics model was established by integrating these four image modalities by using SVM and MOGONET. Compared with the traditional machine learning classification method, MOGONET utilizes the advantage of each imaging modality and considers the correlations among samples analyzed by similarity networks of graph convolutional networks (GCN) to obtain imaging modality-specific GCNs. Next, the modality-specific GCNs were fed into a cross-image modality discovery tensor to explore the cross-image modality correlation at label space. Then, the View Correlation Discovery Network (VCDN) was used for effective multi-image modality integration to obtain the final prediction with the cross-image modality discovery tensor. A nomogram for cervical LNM prediction was developed based on clinical risk factors as well as the prediction performed using MOGONET by stepwise multivariate logistic regression analyses.

### 2.7. Statistical Analysis

Continuous variables are presented as mean ± standard deviation values, and categorical variables are shown as counts (percentages). The Chi-square or Fisher's exact test was employed for the comparison of categorical variables. The *t*-test or Mann–Whitney test was employed for the comparison of continuous variables depending on data distribution. The performance of the LNM prediction models was evaluated by ROC analysis, and the sensitivity (SEN), specificity (SPE), positive predictive value (PPV), negative predictive value (NPV), accuracy (ACC), and AUC were recorded. The nomogram performance was also evaluated using ROC analysis. DeLong's test was applied for comparison between ROC curves, and net reclassification improvement (NRI) and integrated discrimination improvement (IDI) were calculated. The Hosmer–Lemeshow test was used to assess the nomogram's goodness-of-fit. Finally, the decision curve analysis (DCA) was performed for evaluating the nomogram's clinical utility. IBM SPSS Statistics 26.0 (IBM Corp, Armonk, NY, USA), R software (version4.1.3; https://www.r-project.org/), and Python (version 3.5.6; https://www.python.org/) were used for all the statistical analyses. The “mRMR” algorithm in the “mRMRe” package was used to employ the maximum relevance minimum redundancy algorithm to initially screen the radiomics features. The best feature cohort was selected by the “glmnet” algorithm in the “glmnet” package. ROC analysis was conducted based on the “pROC” package to evaluate effectiveness. The “caliplot2” function in the “ModelGood” package was applied to plot the calibration curves, and decision curves were plotted using on “rmda” package. The MOGONET algorithm we used was shown in the literature. A two-tailed*p* value of <0.05 was considered, as statistical significance.

## 3. Results

### 3.1. Patient Characteristics

The study population included 133 patients with PTC (40 males, 93 females; age 44.69 ± 13.50 years; age range, 13–77 years). The incidence rate of cervical LNM was 56.39% (75/133). The patients' detailed clinical characteristics are summarized in Table 1. None of the clinical characteristics was significantly different between the training and validation cohorts. The associations between clinical characteristics and LNM in the training and validation cohorts are presented in Table 2. The non-LNM and LNM groups in both cohorts showed no significant differences in age, sex, and the number of lesions. Tumor diameter was larger in the LNM group in both cohorts. The LNM group showed a greater frequency of bilateral and multifocal PTCs (training cohort: *p* < 0.05; validation cohort: *p* > 0.05). In both cohorts, the LNM group had a markedly higher frequency of thyroid contour protrusion with a more poorly defined tumor margin (*p* < 0.001). Similarly, in both cohorts, microcalcification was more commonly seen in the LNM group (*p* < 0.05). The training cohort showed significant differences in the incidence of an aspect ratio of >1 between the two groups (*p*=0.004).

### 3.2. Performance of the Radiomics Models

**Figure 1:**
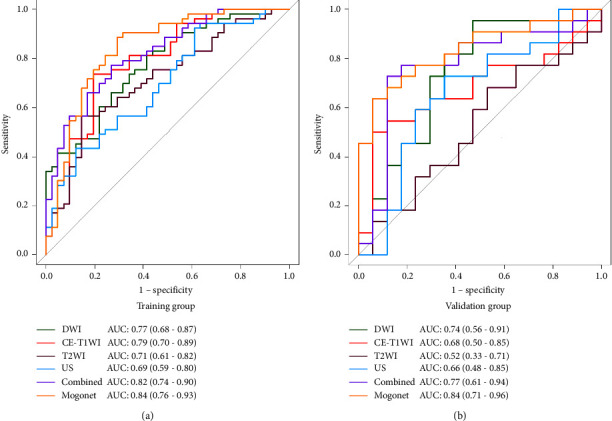
Comparison of ROC curves of the six models for LNM prediction in the cohort. The AUC of the MOGONET model is higher than those of the other five models in both the training (a) and validation (b) cohorts.

After the interobserver ICC analysis, high-throughput features were extracted, 740 from US images, 1045 from T2WI images, 1045 from DWI images, and 785 from CE-T1WI images. Eventually, the nine most predictive subset features were separately selected from US, DWI, T2WI, and CE-T1WI images, and 18 subset features were selected from combined images. Feature importance was evaluated. Supplementary Figure 2 presents the selected features and their importance.  Figure 1 shows the ROC curves for the radiomics models in distinguishing the LNM group from the non-LNM group in both cohorts. The combined radiomics model showed better performance than the other four radiomics models in both cohorts. The AUC, ACC, SEN, SPE, PPV, and NPV of the six models are detailed in Table 3.

### 3.3. Performance of MOGONET Model

**Figure 2:**
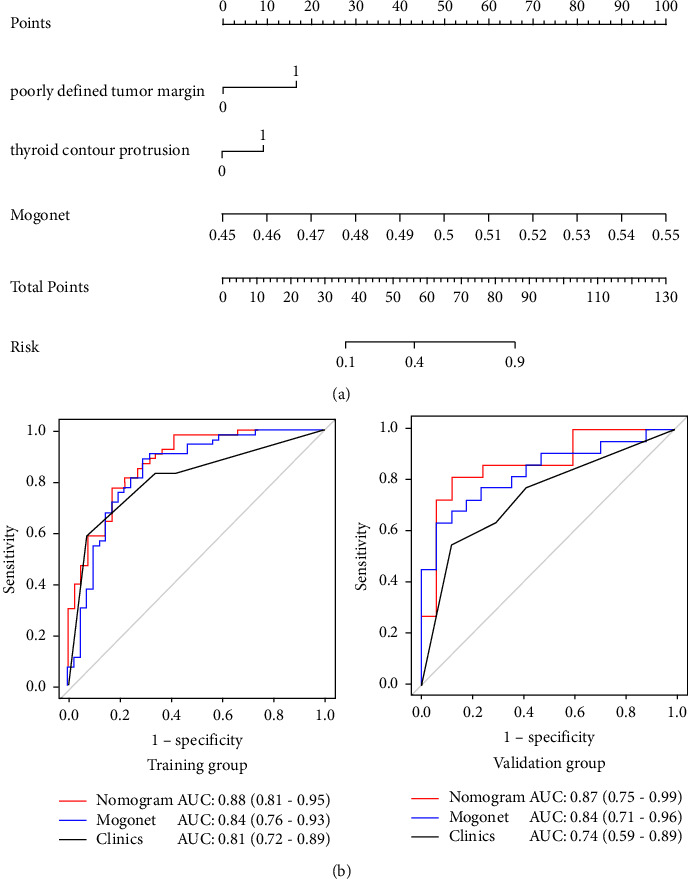
Comparison between the clinical and multimodal radiomics nomograms. (a) The clinical multimodal radiomics nomogram incorporated the MOGONET signature into the clinical model. (b) The ROC curves for nomogram evaluation in the training and validation cohorts. The red, blue, and black lines are the curves of the clinical multimodal radiomics nomogram, MOGONET, and the clinical model, respectively. ROC, receiver operating characteristic; AUC, area under the receiver operating characteristic curve.

To boost the classification performance of the multiradiomics model, we further used MOGONET as a classifier. Compared with the combined radiomics model, the MOGONET model performed better in the training (AUC = 0.84 vs. 0.82) and validation (AUC = 0.84 vs. 0.77) cohorts (Figure 2), according to the ROC analysis. Notably, MOGONET improved the diagnostic specificity. The specificity of the MOGONET, CE-T1WI-radiomics, T2WI-radiomics, DWI-radiomics, and combined radiomics models were 0.71 vs. 0.94, 0.74 vs. 0.53, 0.85 vs. 0.59, 0.51 vs. 0.53, 0.39 vs. 0.18, and 0.73 vs. 0.67 in the training and validation cohorts, respectively (Table 3).

### 3.4. Performance of the Predictive Nomogram

**Figure 3:**
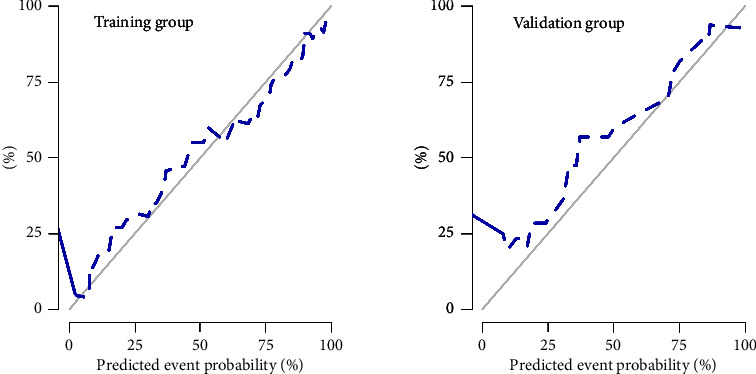
Calibration curves of the clinical multimodal radiomics nomogram in the training and validation cohorts. The Hosmer–Lemeshow test showed no significant difference (*p* > 0.05) in both cohorts.

Multivariable logistic regression analysis indicated that poorly defined tumor margin [OR (95% CI): 3.56(1.39–19.15), *p* = 0.008], thyroid contour protrusions [OR (95% CI): 3.18 (1.15–8.78), *p* = 0.026], and MOGONET [OR (95% CI): 7.72 (3.05–19.58), *p* ≤ 0.01] were independent clinical LNM predictors among patients with PTC (*p* < 0.05) (Table 4). Therefore, we constructed a nomogram for LNM prediction using these predictors (Figure 2(a)). The ROC analysis showed that the AUC (95% CI) of the nomogram was 0.88 (0.81–0.95) and 0.87 (0.75–0.99) in the training and validation cohorts, respectively (Figure 2(b)), which was higher than those associated with the clinical model. This finding suggests that this nomogram had the good discriminative ability. The Hosmer–Lemeshow test showed good agreement between the fitting and observed values in both cohorts (all *p* > 0.05) (Figure 3). DeLong's test showed that the AUCs were not significantly different between the nomogram and clinical model in both cohorts (*p* > 0.05). There were significant differences in NRI and IDI between the two groups. NRI (95% CI) and IDI (95% CI) were 0.96 (0.60–1.31; *p* ≤ 0.01) and 0.14 (0.07–0.21; *p* ≤ 0.01) in the training cohort and were 0.71 (0.14–1.29; *p* = 0.015) and 0.17 (0.0471–0.2891; *p* = 0.006) in the validation cohort.

**Figure 4:**
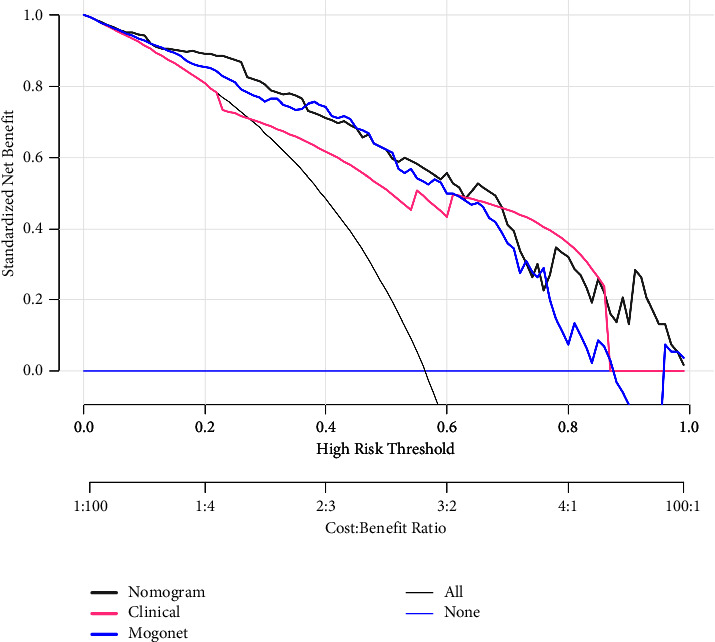
Decision curve analysis of the clinical multimodal radiomics nomogram. The dark blue, blue, pink, and gray lines are the decision curves of our nomogram, MOGONET, the clinical model, and all PTC patients with LNM who received treatment, respectively. The light blue line is the decision curve of all PTC patients without LNM who did not receive treatment. The decision curves show that the clinical multimodal radiomics nomogram to predict LNM in patients with PTC provides a greater benefit than does the clinical model.

DCA of the nomogram and the clinical model was performed to determine whether the nomogram can improve the net benefit for patients. The DCA results indicated that the nomogram had a greater net benefit than the clinical models when the threshold probability was between 0 and 0.7 (Figure 4).

## 4. Discussion

In patients with PTC, cervical LNM indicates local recurrence risk and poor prognosis. We aimed to develop a useful tool based on multiradiomics data for predicting cervical LNM preoperatively. To this end, we constructed four radiomics models using T2WI, DWI, CE-T1WI, and US images, and one linear combination model based on multimodal images using a traditional classification algorithm (SVM). We also used MOGONET for classifier multiradiomics data and compared its performance with other models based on SVM. MOGONET showed better predictive performance (AUC >0.8 in both cohorts) than did the other models, suggesting that multiradiomics model could be invaluable for predicting cervical LNM in PTCs. The DCA also validated the potential applicability of the nomogram incorporating the MOGONET model and clinical risk factors. This approach could be helpful for early medical management and avoid overdiagnosis and overtreatment in PTC.

Although the prognosis of PTC is much better than those of many other cancers, cervical LNM occurs in 30%–80% of the patients with PTC. Cervical LNM is an important consideration in surgical procedures and clinical management for patients with PTC. Nodal metastases most commonly occur at cervical level VI. The ATA guidelines recommend therapeutic lymph node dissection for cN1 disease in cases of PTC. However, the role of prophylactic CCND for cN0 disease is extremely controversial. Some studies have suggested that prophylactic CCND could reduce local recurrence while improving the accuracy of recurrence risk assessment. In contrast, other studies have demonstrated that prophylactic CCND offers no clear benefit for the long-term outcome and is associated with a higher potential for complications, including hemorrhage and injuries to the posterior recurrent nerve. These findings highlight the need to develop a method showing improved accuracy for preoperative LNM prediction while more accurately identifying high-risk patients.

Although US is the preferred imaging modality for assessing thyroid lesions and cervical lymph nodes, US cannot adequately reveal the central region and shows limited ability to identify central cervical LNM. Moreover, operator-related differences substantially influence the accuracy of US-based diagnoses of cervical LNM.

Several recent studies on clinical prediction models have shown that complex echo patterns, posterior region homogeneity, microcalcifications, extrathyroidal extension, capsule contact, age ≤45 years, and tumor size >1.0 cm were independent indicators of cervical LNM among patients with PTC. However, these predictors are subjective and showed variable sensitivity and specificity in predicting cervical LNM among patients with PTC.

In comparison with conventional image analysis, radiomics features provide objective information about the lesion. Radiomics signatures have been shown to be useful for predicting LNM and prognosis in cancer studies. Moreover, radiomics has been used to predict LNM among patients with PTC. Liu et al. established a US-based radiomics model to predict cervical LNM among patients with PTC and reported AUCs of 0.78 in the training cohort and 0.73 in the validation cohort. A nomogram based on shear-wave elastography (SWE) radiomics also showed good calibration and discrimination ability (AUC = 0.83 in the test), demonstrating that SWE radiomics signature is a useful biomarker for cervical LNM prediction among patients with PTC [25]. Yu et al. [20] proposed the transfer learning radiomics for the LNM prediction model of PTC, and the model achieved an AUC of 0.93 and yielded more benefits than other methods. In comparison with qualitative CT image features, the radiomics signature of dual-energy CT iodine maps performed better in the preoperative diagnosis of cervical LNM of PTC [26]. In our previous study, we also demonstrated that radiomics based on multiparameter MRI could adequately predict cervical LNM in PTC patients (AUC = 0.83 in the test cohort) [22].

The radiomics studies described above were conducted on the basis of single-modality medical images. As far as we know, no previous study has compared the performance of radiomics models based on different or multiple imaging modalities. Therefore, we constructed radiomics models based on US and MR images, including DWI, T2WI, and CE-T1WI sequences, and compared their predictive performance. In the validation cohort, the AUCs of the DWI-radiomics, T2WI-radiomics, CE-T1WI-radiomics, and US-radiomics models were 0.74, 0.52, 0.68, and 0.66, respectively. Previous studies have shown that multiparametric MRI combination can improve diagnostic efficacy [22]. Liu et al. also showed that *B*-mode images together with strain elastography improved cervical LNM prediction, with the AUC increasing from 0.81 for *B*-mode images to 0.90 for multimodality images. Multiomics data can yield more accurate clinical outcome predictions than single-type omics data. Our results corroborated these findings by showing that the combined radiomics model constructed from the linear fusion of all features had a better performance than the unimodular model in both training and validation cohorts.

The existing classification methods based on supervised data integration includes the strategies based on feature concatenation and ensembles. In the methods based on concatenation, different types of omics data are integrated by directly concatenating the input data features to train the classification model. In contrast, predictions from different classifiers are integrated into ensemble-based methods. However, these methods did not account for correlations among different types of omics data. MOGONET, a new multiomics integrative algorithm was proposed recently. In comparison with KNN, SVM, and the other seven existing integration methods, MOGONET achieved the best performance in all classification tasks on three databases. In addition, MOGONET was superior to the latest supervised multiomics integration methods. Exploration of cross-omics label correlations through VCDN and integration of classification results from different types of omics data yielded consistent improvements in classification performance.

In this study, MOGONET models trained with four types of radiomics data achieved better performance than combined radiomics model constructed by SVM. The AUCs (95% CI) of MOGONET model in the training and validation cohorts were 0.84 (0.76–0.93) and 0.84 (0.71–0.96), respectively, while the corresponding AUCs (95% CI) of the combined radiomics model was 0.82 (0.74–0.90) and 0.77 (0.61–0.94), respectively. Notably, MOGONET models improved the specificity of predicting LNM.

The logistic regression analyses suggested that in PTC patients, poorly defined margins, thyroid contour protrusion, and MOGONET scores were independent risk factors for LNM. The AUC of the nomogram was higher than that of clinical models. Although DeLong's test showed no significant differences between the ROC curves of the clinical models and the nomogram (*p* > 0.05), the small sample size may have influenced this finding. In contrast, NRI and IDI associated with the nomogram were significantly higher than those associated with the clinical models in both cohorts (*p* < 0.05), indicating an improved prediction probability. DCA analysis showed a net benefit of the nomogram.

The study had several limitations. First, the data were obtained from a single centre and lacked external validation. Second, the sample size was small, and the prognostic value of the findings should be further validated in the future. Third, since this was a retrospective study, LN status was evaluated on the basis of postoperative pathology. The largest long-axis cross-section image on the US was dependent on the operator, and we cannot ensure complete consistency between the largest long-axis cross-section images obtained using US and MRI. Thus, selection bias was inevitable and may have affected the results. Finally, US examinations were not performed on the same machine and by the same radiologist. The resultant inconsistencies in inspection parameters may have affected the accuracy of the results and need to be verified with large-sample multicentre data.

## 5. Conclusions

In conclusion, the MOGONET model integrated multiradiomics performed better in LNM prediction than radiomics constructed from single imaging modality data. This noninvasive clinical multimodal radiomics nomogram may facilitate clinical decision-making for patients showing PTC.

## Figures and Tables

**Table 1:** Clinical characteristics of PTCs in the training and validation cohorts.

Variable	Training cohort	Validation cohort	Statistics	*p* value
Age (years)	44.73 ± 13.6	44.59 ± 13.41	0.001	0.977
Diameter (cm)	1.27 ± 0.61	1.32 ± 0.68	0.42	0.526
Gender			0.279	0.598
Female	67 (71.28%)	26 (66.67%)		
Male	27 (28.72%)	13 (33.37%)		
Location			3.932	0.281
Right lobe	34 (36.17%)	14 (35.90%)		
Left lobe	47 (50.0%)	22 (56.41%)		
Isthmus	5 (5.32%)	3 (7.69%)		
Bilateral	8 (8.51%)	0 (0%)		
Number_of_lesions			6.11	0.137
1	71 (75.53%)	37 (94.87%)		
2	17 (18.09%)	2 (5.13%)		
3	3 (3.19%)	0 (0%)		
4	2 (2.13%)	0 (0%)		
5	1 (1.06%)	0 (0%)		
Tumor margin on T1WC+			1.91	0.167
Well defined	36 (38.3%)	20 (51.28%)		
Poorly defined	58 (61.70%)	19 (48.72%)		
Thyroid contour protrusion sign on T1WC+			0.524	0.469
Absent	57 (60.64%)	21 (53.85%)		
Present	37 (39.36%)	18 (46.15%)		
Aspect ratio on US imaging			0.696	0.404
<1	63 (67.02%)	29 (74.36%)		
>1	31 (32.98%)	10 (25.64%)		
Microcalcification			0.115	0.735
Absent	44 (46.81%)	17 (43.59%)		
Present	22 (53.19%)	22 (56.41%)		
MOGONET	0.53 ± 0.50	0.56 ± 0.50	0.545	0.462

PTC, papillary thyroid carcinoma; LNM, lymph node metastasis. *p* values of <0.05 were considered to indicate statistical significance.

**Table 2:** Clinical characteristics of PTCs in the LNM and non-LNM groups in the training and validation cohorts.

Variable	Training cohort		Statistics	*p* value	Validation cohort		Statistics	*p* value
	Non-LNM	LNM			Non-LNM	LNM		
Age (years)	47.90 ± 13.75	42.28 ± 13.09	1.876	0.061	46.12 ± 13.63	43.41 ± 13.44	0.62	0.539
Diameter (cm)	1.07 ± 0.97	1.20 ± 0.79	−2.097	0.036^*∗*^	0.97 ± 0.39	1.46 ± 0.65	−3.654	<0.001^*∗*^
Gender			0.316	0.574			2.25	0.318
Female	28 (68.29%)	39 (73.58%)			13 (76.47%)	13 (59.09%)		
Male	13 (31.71%)	14 (26.42%)			4 (23.53%)	9 (40.91%)		
Location			9.87	0.024^*∗*^			2.33	0.374
Right lobe	16 (39.02%)	18 (33.96%)			6 (35.29%)	8 (36.36%)		
Left lobe	24 (58.54%)	23 (43.40%)			11 (64.71%)	11 (50.00%)		
Isthmus	1 (2.44%)	4 (7.55%)			0 (0.00%)	3 (13.64%)		
Bilateral	0 (0.00%)	8 (15.09%)						
Number_of_lesions			7.08	0.057				0.495
1	36 (87.80%)	35 (66.04%)			17 (100%)	20 (90.91%)		
2	4 (9.76%)	13 (24.53%)			0 (0.00%)	2 (9.09%)		
3	0 (0.00%)	3 (5.66%)						
4	1 (2.44%)	1 (1.89%)						
5	0 (0.00%)	1 (1.89%)						
Tumor margin on T1WC+			23.366	<0.001^*∗*^			46.667	<0.001^*∗*^
Well defined	27 (65.85%)	9 (16.98%)			14 (82.35%)	2 (9.09%)		
Poorly defined	14 (34.15%)	44 (83.02%)			3 (17.65%)	20 (90.91%)		
Thyroid contour protrusion sign on T1WC+			18.628	<0.001^*∗*^			101.333	<0.001^*∗*^
Absent	35 (85.37%)	22 (41.51%)			16 (94.12%)	3 (13.64%)		
Present	6 (14.63%)	31 (58.49%)			1 (5.88%)	19 (86.36%)		
Aspect ratio on US imaging							7.467	0.106
<1	34 (82.93%)	29 (54.72%)	8.323	0.004^*∗*^	16 (94.12%)	15 (68.18%)		
>1	7 (17.07%)	24 (45.28%)			1 (5.88%)	7 (31.82%)		
Microcalcification							5.143	0.025^*∗*^
Absent	27 (65.85%)	17 (32.08%)	10.593	0.001^*∗*^	12 (70.59%)	7 (31.82%)		
Present	14 (34.15%)	36 (67.92%)			5 (29.41%)	15 (68.18%)		
MOGONET	0.49 ± 0.01	0.51 ± 0.01	−6.609	<0.001^*∗*^	0.49 ± 0.01	0.51 ± 0.01	−3.569	<0.001^*∗*^

PTC, papillary thyroid carcinoma; LNM, lymph node metastasis. *p* values of <0.05 were considered to indicate statistical significance.

**Table 3:** Quantitative indices pertaining to the six models for data from two cohorts.

Model	Cohorts	AUC (95% CI)	ACC	SEN	SPE	PPV	NPV
MOGONET	Training	0.84 (0.76–0.93)	0.82	0.89	0.71	0.80	0.83
	Validation	0.84 (0.71–0.96)	0.77	0.76	0.94	0.88	0.74

Combined-radiomic	Training	0.82 (0.74–0.90)	0.80	0.85	0.74	0.77	0.83
	Validation	0.77 (0.61–0.94)	0.77	0.93	0.67	0.64	0.94

DWI-radiomic	Training	0.74 (0.56–0.91)	0.72	0.89	0.51	0.70	0.78
	Validation	0.74 (0.56–0.91)	0.74	0.91	0.53	0.71	0.82

CE-T1WC-radiomic	Training	0.79 (0.70–0.89)	0.77	0.74	0.80	0.83	0.70
	Validation	0.68 (0.50–0.85)	0.64	0.73	0.53	0.67	0.60

T2WI-radiomic	Training	0.71 (0.61–0.82)	0.69	0.57	0.85	0.83	0.60
	Validation	0.52 (0.33–0.71)	0.51	0.45	0.59	0.59	0.45

US-radiomic	Training	0.69 (0.59–0.80)	0.69	0.92	0.39	0.66	0.80
	Validation	0.66 (0.48–0.85)	0.59	0.91	0.18	0.59	0.60

AUC, area under the receiver operating characteristic curve; ACC, accuracy; SEN, sensitivity; SPE, specificity; PPV, positive predictive value; NPV, negative predictive value; CI, confidence interval.

**Table 4:** Univariate and multivariate analyses of the preoperative predictors of LNM of PTCs in the training cohort.

Variable	Univariate analysis		Multivariate analysis	
	OR (95% CI)	*p* value	OR (95% CI)	*p* value
Age, <45 years	1.76 (0.77–4.02)	0.050	—	—
Diameter, <1 cm	2.0 (0.86–4.67)	0.026^*∗*^	—	—
Gender	0.77 (0.32–1.9)	0.574	—	—
Location	1.85 (1.06–3.2)	0.029^*∗*^	—	—
Number_of_lesions	2.16 (0.99–4.74)	0.054	—	—
Tumor margin on T1WC+	9.43 (3.59–24.74)	<0.001^*∗*^	3.56 (1.39–19.15)	0.008^*∗*^
Thyroid contour protrusion sign on T1WC+	8.22 (2.95–22.89)	<0.001^*∗*^	3.18 (1.15–8.78)	0.026^*∗*^
Aspect ratio on US imaging	4.02 (1.51–10.68)	0.005^*∗*^	—	—
Microcalcification	4.08 (1.72–9.71)	0.001^*∗*^	—	—
MOGONET	14.03 (5.98–32.95)	<0.001^*∗*^	7.72 (3.05–19.58)	<0.001^*∗*^

PTC, papillary thyroid carcinoma; LNM, lymph node metastasis; CI, confidence interval; OR, odds ratio; ^*∗*^*p* < 0.05.

## Data Availability

The data used to support the findings of this study are available from the corresponding author upon request.

## Supplementary Materials

Supplementary Materials Supplementary Figure 1. Flow chart of the patient selection process. PTC, papillary thyroid carcinoma; MRI, magnetic resonance imaging; US, ultrasound. Supplementary Figure 2. The final selected features extracted from CE-T1, T2WI, DWI, US, and US combined MRI (combined radiomics) models to distinguish LNM from non-LNM patients by using the SVM method. T2WI, T2-weighted imaging; DWI, diffusion-weighted imaging; CE-T1, T1-weighted contrast-enhanced imaging; US, ultrasound. Supplementary Table 1. Magnetic resonance sequence parameters.
