# Supplementary material for: Preoperative Cervical Lymph Node Metastasis Prediction in Papillary Thyroid Carcinoma: A Noninvasive Clinical Multimodal Radiomics (CMR) Nomogram Analysis
Source: J Oncol. 2023 Mar 9;2023:3270137. doi: 10.1155/2023/3270137 (PMC10019962; doi:10.1155/2023/3270137)
Supplement: Supplementary Materials — Supplementary Figure 1. Flow chart of the patient selection process. PTC, papillary thyroid carcinoma; MRI, magnetic resonance imaging; US, ultrasound. Supplementary Figure 2. The final selected features extracted from CE-T1, T2WI, DWI, US, and US combined MRI (combined radiomics) models to distinguish LNM from non-LNM patients by using the SVM method. T2WI, T2-weighted imaging; DWI, diffusion-weighted imaging; CE-T1, T1-weighted contrast-enhanced imaging; US, ultrasound. Supplementary Table 1. Magnetic resonance sequence parameters. [file 3270137.f1.zip › Supplementary figure 2.pdf]

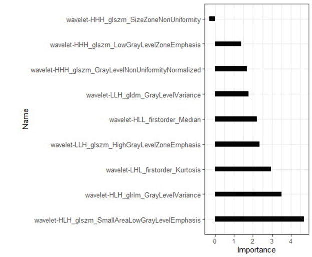

CE-T1WI radiomics features

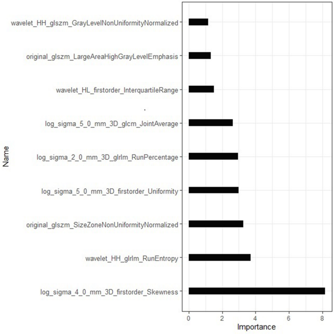

US radiomics features

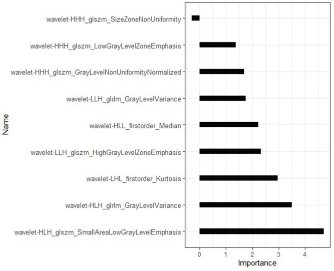

T2WI radiomics features

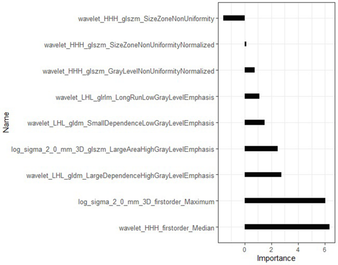

DWI radiomics features

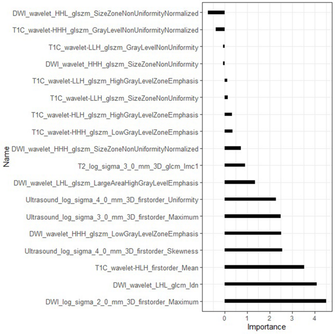

Combined radiomics features
