# Supplementary material for: Preoperative Cervical Lymph Node Metastasis Prediction in Papillary Thyroid Carcinoma: A Noninvasive Clinical Multimodal Radiomics (CMR) Nomogram Analysis
Source: J Oncol. 2023 Mar 9;2023:3270137. doi: 10.1155/2023/3270137 (PMC10019962; doi:10.1155/2023/3270137)
Supplement: Supplementary Materials — Supplementary Figure 1. Flow chart of the patient selection process. PTC, papillary thyroid carcinoma; MRI, magnetic resonance imaging; US, ultrasound. Supplementary Figure 2. The final selected features extracted from CE-T1, T2WI, DWI, US, and US combined MRI (combined radiomics) models to distinguish LNM from non-LNM patients by using the SVM method. T2WI, T2-weighted imaging; DWI, diffusion-weighted imaging; CE-T1, T1-weighted contrast-enhanced imaging; US, ultrasound. Supplementary Table 1. Magnetic resonance sequence parameters. [file 3270137.f1.zip › Supplementary Table 1 (1).docx]

Supplementary Table 1. Magnetic Resonance Sequences Parameters.

|  | Axial T2WI | Axial DWI | Axial CE-T1WI |
| --- | --- | --- | --- |
| TE, ms | 85 | Minimum | 1.7 |
| TR, ms | 3000 | 6550 | 5.7 |
| FOV, cm | 14 | 14 | 14 |
| Thickness, mm | 4 | 4 | - |
| Spacing, mm | 0.5 | 0.5 | - |
| Matrix | 320 × 224 | 128 × 128 | 192 × 256 |
| NEX | 4 | 4 | 1 |

Note: T2WI,T2-weighted imaging ; DWI, diffusion-weighted imaging; CE-T1WI,contrast-enhanced T1-weighted imaging; TE, echo time; TR, repetition time. FOV, field of view.
